# Distinguishing paranormal belief and paranormal involvement: canonical correlation analysis of positive psychological functioning

**DOI:** 10.3389/fpsyg.2026.1821311

**Published:** 2026-08-05

**Authors:** Neil Dagnall, Kenneth Graham Drinkwater, Zoe Danielle Hughes, Robert C. Dempsey, Stephen Walsh, Álex Escolà-Gascón, Andrew Denovan

**Affiliations:** 1 School of Psychology, Manchester Metropolitan University, Manchester, United Kingdom; 2 School of Psychology, Liverpool John Moores University, Liverpool, United Kingdom; 3 Department of Quantitative Methods and Statistics, Universidad Pontificia Comillas, Madrid, Spain

**Keywords:** individual differences, mental health, paranormal belief, paranormal involvement, positive psychology, wellbeing

## Abstract

**Background:**

Psychological focus on supernatural credence has overlooked how behavioural engagement with the paranormal relates to wellbeing. Addressing this gap, this study distinguished between paranormal belief (PB; cognitive credence) and paranormal involvement (PI; behavioural engagement) and examined multivariate associations with positive psychological functioning. It further investigated whether PB and PI represented discrete yet related dimensions of personal paranormal salience (i.e., perceived significance).

**Method:**

A UK sample (*N* = 2,011; *M*age = 50.18 years) completed self-report measures assessing PB (Traditional Paranormal Belief and New Age Philosophy), PI, hope (agency and pathways), optimism, positive emotion, hedonic and eudaimonic wellbeing, and mental toughness. Canonical correlation analysis (CCA) examined shared variance between paranormal variables and positive psychological indicators.

**Results:**

CCA revealed two significant canonical functions. Function 1, defined by PB, aligned moderately with optimism and hope agency, indicating an attitudinal–motivational profile. Function 2, characterised by PI, showed stronger associations with hedonic and eudaimonic wellbeing, hope pathways, and mental toughness, reflecting a proactive and resilient psychological formation. Although PB and PI were positively correlated, they contributed to distinct multivariate patterns. Collectively, findings indicated that distinguishing PB from PI revealed functionally distinct psychological profiles, consistent with the theoretical conceptualisation of paranormal salience (PS) as a higher-order framework encompassing belief and engagement.

**Conclusion:**

PB and PI can be conceptualised as functionally distinct yet complementary components of PS. In terms of wellbeing, behavioural involvement aligned more consistently with positive psychological functioning.

## Introduction

Within psychological research, the Revised Paranormal Belief Scale (RPBS) is the preponderant measure of supernatural credence (Tobacyk, 2004). The instrument is an improved version of the Paranormal Belief Scale (PBS; Tobacyk and Milford, 1983), which developed in response to burgeoning public interest in the preternatural during the 1970s and 1980s. The PBS/RPBS operationalizes paranormal belief (PB) as the endorsement of notions that are scientifically inexplicable and incompatible with normative reality (Irwin, 2009). Explicitly, belief in phenomena, which violate the basic limiting principles of science (i.e., physical causality, spatiotemporal continuity governed by invariant laws, and the exclusion of immaterial entities as causal agents) (Broad, 1949).

To develop the PBS, Tobacyk and Milford (1983) combined items from existing instruments with new material, then factor analysis identified seven factors: Traditional Religious Belief (i.e., orthodox Christian faith), Psi (i.e., existence of telepathy and psychokinesis), Witchcraft (i.e., efficacy of magic), Superstition (i.e., customary folklore and/or omens), Spiritualism (i.e., the non-physical soul), Extraordinary Life Forms (i.e., existence of cryptids or unrecognized biological entities), and Precognition (i.e., prediction or perception of future events). These dimensions defined the structure of PB and PBS content.

The PBS by progressing the study of supernatural credence from anecdotal observation to psychometric measurement established a robust methodology for quantifying and conceptualizing supernatural credence in Western societies. The R-PBS (Tobacyk, 2004) arose from enhancement of PBS measurement precision and cross-cultural validity. Explicitly, modifications consolidated factorial structure by removing cross-loading items, rewording subscales to improve clarity, and heightening measurement sensitivity, by shifting from five to seven response options.

Despite these refinements, critics continued to question the factorial composition of the RPBS because Tobacyk and Milford (1983) failed to prescribe a strict, orthogonal structure. Assessment resulted in the forwarding of alternative models. Illustratively, Lawrence (1995) reported an oblique five-factor model (i.e., Traditional Religious Belief, Psychic Beliefs, Superstition, Witchcraft, and Anomalous Natural Phenomena), and Hartman (1999) proposed a four-factor solution (Psi, Traditional Religion, Superstition, and Witchcraft).

Acknowledging factorial ambiguity and the tendency of investigators to use total rather than subscale scores, Drinkwater et al. (2017) reassessed RPBS structure. Analysis identified a superior bifactor solution that located the original subfactors within an overarching general factor. This model specified that global supernatural orientation explained the majority of variance. Since PB is heterogeneous, this outcome, although statistically robust, was theoretically simplified because similar total scores can arise from different configurations of belief (Beck and Miller, 2001; Drinkwater et al., 2024a; Irwin, 1997). Consequently, reliance on total scores may obscure psychologically meaningful differences in the functional role that distinct belief profiles serve for individuals. This concern is not that overall scores lack utility, but that they may conceal functionally distinct patterns of belief that become important when the objective is to understand psychological adaptation rather than overall endorsement.

To extend understanding beyond overall belief intensity, research should consider the adaptive functions that may underlie different paranormal orientations (Connors and Halligan, 2022; Irwin, 2004; Irwin and Watt, 2007). One approach to examining these potential functional differences is the Lange et al. (2000) two-factor model. This emerged from top-down Rasch purification of the RPBS, which removed non-additive items and differentially functioning items. The resulting structure provided a framework for interpreting paranormal belief according to potential psychological function rather than solely phenomenological content.

Factor 1, Traditional Paranormal Belief (TPB) focuses on culturally established constructs rooted in religion or folklore, such as the devil, heaven and hell, witchcraft, and premonition. Operationally, TPB affords existential security through these externally grounded systems of meaning and authority (Tobacyk, 1995). This dimension is associated with fear-based interpretations and a mechanistic perception of life, particularly the anxiety that uncontrollable forces govern or influence existence (Houran et al., 2001). This alignment implies that while TPB provides a structured framework for understanding the universe, it concurrently reflects an underlying concern with mitigating the psychological impact of supernatural threats. Consistent with this, research indicates that these belief systems are frequently linked with active coping strategies during times of environmental stress (Keinan, 2002; Lasikiewicz, 2016).

In contrast, factor 2, New Age Philosophy (NAP) centres on modern paranormal capacities and transcendental states, including psi (psychokinesis/precognition), spiritualism, and astral projection. Rather than seeking security through external authority, NAP facilitates a sense of personal agency via experiential and intuitive forms of control (Irwin, 2009). Consequently, this dimension is linked to individualism, perceived self-control, and sensation-seeking tendencies (Houran et al., 2001). While cultural and social factors strengthen TPB, NAP is reinforced through personal experience and subjective validation, marking a fundamental divergence in how these supernatural worldviews are sustained and expressed. The functional divergence of these beliefs is evidenced by the finding that TPB significantly predicted higher distress and lower coping, whereas NAP was not associated with either (Drinkwater et al., 2024b).

Nonetheless, differences in belief composition alone do not capture how discrete worldviews influence everyday interactions. Hence, to distinguish between ideological endorsement of beliefs and their experiential and behavioural expression, the present study included paranormal involvement (PI). PI indexes direct engagement with the supernatural (i.e., self-reported experiences, practitioner consultation, and perceived abilities) and provides an operational measure of experiential reinforcement (Drinkwater et al., 2024a; Houran and Laythe, 2022). This theoretical distinction is important given that TPB is sustained through cultural transmission and external authority (Boudry, 2019; Houran et al., 2001; Willard and Norenzayan, 2013). Thus, the inclusion of PI enables a holistic and ecologically valid examination of how supernatural belief systems and engagement interact in relation to psychological wellbeing (e.g., Goulding, 2005; Houran and Laythe, 2022; Rabeyron and Watt, 2010).

While previous research has examined paranormal belief primarily from cognitive and reasoning perspectives (e.g., Lindeman and Aarnio, 2006; Pennycook et al., 2012) or in relation to psychological distress (Drinkwater et al., 2024b), the present study examines its association with positive psychological functioning, including hope, optimism, and mental toughness. Within this framework, mental toughness functions as an adaptive psychological buffer, enabling individuals to maintain performance under pressure and reframe challenges as developmental opportunities (Clough et al., 2002; Dagnall et al., 2019; Papageorgiou et al., 2018). Incorporating MT allowed the present study to examine whether PI, particularly within the agentic context of NAP, aligned with a hardy disposition. By synthesizing belief systems with behavioural engagement, analysis advanced beyond attitudinal accounts to explore how credence manifests as a lived experience.

To assess these relationships, the current paper employed canonical correlation analysis (CCA). This multivariate technique was selected for its ability to identify principal sources of covariation between paranormal and psychological sets (Boedeker and Henson, 2020). By modelling belief systems (i.e., TPB and NAP), PI, and wellbeing within a shared psychological space, CCA offered a theoretically coherent and ecologically valid method for capturing the complex interplay between supernatural worldviews and psychological functioning (cf. Callaghan and Irwin, 2003).

It was hypothesized that TPB and NAP would demonstrate a significant multivariate association with indicators of positive psychological functioning (H1). Specifically, that belief endorsement would align with adaptive motivational components within the multivariate system. This prediction followed from the functional perspective that PB systems support existential coherence and perceived control, processes central to adaptive motivational functioning (Houran et al., 2001). Furthermore, it was anticipated that PI would exhibit a distinct multivariate association with these psychological indicators, remaining conceptually and statistically separable from belief endorsement (H2). Crucial to the exploration of adaptive traits, we predicted that PI would associate positively with a proactive psychological profile characterized by higher levels of hope (agency and pathways), optimism, eudaimonic and hedonic wellbeing, and mental toughness (H3). Finally, reflecting the complex interplay between ideological conviction and lived experience, CCA was expected to yield multiple significant functions (H4).

## Method

### Participants

#### Sample

The sample comprised 2011 participants (*M*age = 50.18, *SD* = 14.73); 936 males (*M*age = 52.34, *SD* = 14.16), 1,042 females (*M*age = 48.82, *SD* = 14.83), one trans male (age = 62), one trans female (age = 63), 30 non-binary (*M*age = 29.76, *SD* = 4.82), and one preferred not to disclose (age = 50). Participants were recruited through Bilendi, a high-quality data provider (Hajek et al., 2024). The research team requested a general UK-based sample, aged 18 years and over.

### Materials

This study used psychometrically attested self-report measures.

### Paranormal belief

The Revised Paranormal Belief Scale (RPBS) (Tobacyk, 2004) is a 26-item instrument that measures supernatural credence. Items appear as statements (e.g., “Black magic really exists”) and participants record their responses on a 7-point Likert-type scale (1 = strongly disagree to 7 = strongly agree). This study used the two-factor model (Lange et al., 2000), which comprises Traditional Paranormal Beliefs (TPB; 11-items) and New Age Philosophy (NAP; 5-items). TPB assesses endorsement of culturally established constructs (i.e., devil, heaven and hell, witchcraft, and premonition), whereas NAP assesses belief in more recently identified capacities (e.g., psychokinesis and precognition) and transcendental states of consciousness (i.e., astral projection and spirit presence). To calculate dimensions, it was necessary to convert scoring to 0 to 6 then transform raw to Rasch scaled scores.

### Paranormal involvement

Paranormal involvement (PI) is a 3-item scale assessing engagement with supernatural phenomena (Dagnall et al., 2024). Using Yes/No response options, participants indicate whether they have experienced paranormal phenomena, consulted paranormal practitioners, or demonstrated purported paranormal abilities. Responses are summed to produce a total score ranging from 0 to 3, with higher scores indicating greater PI. PI is an established research measure that has been used in published studies (Dagnall et al., 2016; Drinkwater et al., 2021a, 2021b).

The researchers selected this index because the primary objective was to obtain a broad, parsimonious measure of behavioural involvement alongside cognitive belief endorsement (RPBS), rather than assess the frequency, intensity, or phenomenological characteristics of paranormal experiences. The brief dichotomous format reduced respondent burden within an extensive survey battery while effectively capturing the presence of paranormal engagement.

### Hope

The Adult Hope Scale (AHS) (Snyder et al., 1991) is a 12-item instrument that defines hope as a positive motivational state derived from agency and pathways. Agency (Items 2, 9, 10, and 12) represents motivational will and energetic pursuit of goals. Pathways (Items 1, 4, 6, and 8) denotes the cognitive ability to plan viable routes and navigate around obstacles. The remaining four items (3, 5, 7, and 11) serve as fillers. The AHS presents items as statements and participants respond using an 8-point Likert-type scale (1 = definitely false to 8 = definitely true’). Higher subscale totals designate greater agency and pathways.

### Positive and negative experience

The Scale of Positive and Negative Experience (SPANE) (Diener et al., 2010) is a 12-item scale that assesses positive (SPANE-P, e.g., pleasant and joyful) and negative emotional experiences (SPANE-N, e.g., unpleasant and afraid). Items appear as statements and participants record their answers on a Likert-type scale, ranging from “very rarely or never = 1” to “very often or always = 5”. This study used only the positive subscale, higher scores indicating greater positive experience.

### Optimism

The Optimism–Pessimism Short Scale–2 (SOP2) (Kemper et al., 2013; Nießen et al., 2022) assesses dispositional confidence and expectations about the future via endorsement of general statements about optimism and pessimism. Each item contains a brief definition followed by a self-report prompt. In this study, respondents rated their optimism on a 7-point Likert scale ranging from “1 = not at all” to “7 = very”. This study used only the optimism item, with higher scores signifying greater confidence about the future.

### Psychological wellbeing

To assess the multidimensional nature of well-being, this study used the Eudaimonia and Hedonia Scale (Su et al., 2020). Eudaimonic well-being was measured by six items capturing psychological functioning and personal growth. Hedonic well-being was evaluated using five items indexing general happiness and life satisfaction. The instrument presents items as statements and participants record their responses by completing a 7-point Likert-type scale ranging from 1 (strongly disagree) to 7 (completely agree). Higher subscales scores designate greater eudaimonic and hedonic well-being.

### Mental toughness

The Mental Toughness Questionnaire-10 (MTQ10; Dagnall et al., 2019; Papageorgiou et al., 2018) is a 10-item instrument that assesses the degree to which participants possess a positive mindset that enables them to perform effectively under pressure. The MTQ10 captures core variance from the 4/6Cs model (Clough et al., 2002), which comprises Control (perceived influence over life and emotions), Commitment (goal persistence), Challenge (openness to change), and Confidence (self-belief in abilities). Items appear as statements (e.g., “I generally feel that I am a worthwhile person”) and participants record responses on a 5-point Likert scale (1 = Strongly Disagree to 5 = Strongly Agree). Higher scores indicate greater psychological hardiness.

### Reliability

All measures employed have established psychometric integrity (i.e., validity and reliability): RPBS (Drinkwater et al., 2017), PI (Drinkwater et al., 2021a, 2021b), AHS (Snyder et al., 1991), SPANE (Diener et al., 2010), SOP2 (Kemper et al., 2013; Nießen et al., 2022), Eudaimonia and Hedonia Scale (Su et al., 2020), and MTQ10; (Dagnall et al., 2019). In this study, good reliability (omega) existed for multi-item measures (RPBS = 0.96, TPB = 0.87, NAP = 0.94, AHSAgency = 0.88, AHSPathways = 0.86, Positive Emotions = 0.93, Eudaimonia = 0.87, Hedonia = 0.94, MTQ10 = 0.86). Reliability was not computed for single-item measures because such indices require multiple items.

### Procedure

Potential participants received a hyperlink directing them to study details (i.e., aims, objectives, and details). To progress to the survey, it was necessary for participants to record agreement. Consenting participants completed a demographic questionnaire (i.e., age, gender identity, and occupation) then progressed to the survey. Instructions directed participants to proceed through the survey at their own pace. To reduce potential order and carryover effects, Qualtrics randomiser rotated section order across participants. Following survey completion, participants received the study debrief.

Since data collection was cross sectional, the authors implemented methodological strategies to limit common-method variance. Consistent with established guidance, survey instructions highlighted the independence of the constructs under investigation (Podsakoff et al., 2003). This reduced the likelihood of uniform responding by creating psychological separation between sections (Krishnaveni and Deepa, 2013).

In addition, to minimise social desirability effects and evaluation apprehension, the study briefs informed participants that responses should reflect personal views and that there were no correct responses. Researchers report that this approach promotes open, authentic responding (Denovan et al., 2024, 2025).

### Ethics statement

Ethical approval (Project ID, 79268) was granted by the Health and Education Research Ethics and Governance Committee at Manchester Metropolitan University.

## Results

The researchers conducted analyses in IBM SPSS Statistics 31. Prior to canonical correlation analysis (CCA), the dataset was examined for missing values, distributional assumptions, and patterns of intercorrelations among variables. No missing values were identified, eliminating the need for imputation or other missing-data handling procedures. Descriptive statistics (raw scale means, standard deviations, and zero-order correlations) appear in Table 1.

**Table 1:** Means, standard deviations, and Pearson correlations among study variables.

Variable	*M*	*SD*	1	2	3	4	5	6	7	8	9	10
1. NAP	22.72	6.75										
2. TPB	23.75	6.58	0.81**									
3. INVOL	0.60	0.86	0.36**	0.35**								
4. OPT	4.51	1.54	0.16**	0.18**	0.14**							
5. EHStotal	29.49	6.65	0.05*	0.09**	0.13**	0.62**						
6. HEDtotal	43.56	8.03	−0.06**	−0.01	0.10**	0.50**	0.79**					
7. SPANEpos	21.40	4.80	0.04	0.08**	0.06**	0.58**	0.71**	0.64**				
8. HA	21.76	5.60	0.08**	0.09**	0.10**	0.56**	0.71**	0.67**	0.66**			
9. HP	22.52	4.78	0.05*	0.09**	0.15**	0.47**	0.62**	0.60**	0.58**	0.78**		
10. MT	33.65	6.99	−0.08**	−0.05*	0.01	0.54**	0.67**	0.71**	0.65**	0.68**	0.62**	

NAP, new age philosophy; TPB, traditional paranormal belief; INVOL, paranormal involvement; OPT, optimism; EHStotal, Eudaimonic wellbeing; HEDtotal, hedonic wellbeing; SPANEpos, positive emotion; HA, hope agency; HP, hope pathways; MT, mental toughness. *p < 0.05, **p < 0.01.

Pearson correlations revealed a strong association between New Age Philosophy (NAP) and Traditional Paranormal Belief (TPB), *r* = 0.81, *p* < 0.001, indicating substantial shared variance. This degree of association was theoretically anticipated and accords with previous research demonstrating that TPB and NAP represent related components of a broader paranormal belief orientation (Lange et al., 2000; Houran et al., 2001). Although these dimensions share considerable variance, previous research has shown that they exhibit distinct patterns of criterion validity, demonstrating differential relationships with external psychological variables (Houran et al., 2001). Accordingly, the shared variance is interpreted as reflecting a common higher-order belief orientation, while the unique variance retained by each dimension is considered theoretically meaningful and consistent with their functional differentiation. Consequently, both dimensions were retained as separate indicators.

Since CCA models multivariate shared covariance rather than estimating unique regression coefficients, interpretation focused on canonical structure coefficients and the relative contribution of each variable to the canonical functions rather than independent predictive effects. CCA was the most appropriate analytic approach because the objective was to characterise multivariate patterns of shared covariance between paranormal and positive psychological variables, rather than test a prespecified latent variable or structural model as would typically be undertaken using structural equation modelling.

NAP and TPB were moderately associated with Paranormal Involvement (PI) (*rs* = 0.35–0.36, *ps* < 0.001). Regarding psychological functioning, NAP and TPB exhibited a bifurcated relationship with wellbeing. Specifically, dimensions showed small but significant positive associations with Optimism, Hope, and Eudaimonic Wellbeing (*rs* = 0.05–0.18), and were inversely related to Hedonic Wellbeing and Mental Toughness (*rs* = −0.06 to −0.08). PI demonstrated consistent positive associations with Optimism, Wellbeing, Positive Emotion, and Hope (*rs* = 0.06–0.15), and no relationship with Mental Toughness.

Positive psychological variables exhibited strong intercorrelations, with Optimism, Eudaimonic Wellbeing, Hedonic Wellbeing, Positive Emotion, Hope, and Mental Toughness demonstrating moderate-to-strong positive associations (*rs* = 0.47–0.79, *ps* < 0.001). This multivariate convergence indicated a coherent positive psychological functioning cluster. Comparatively, while PB dimensions demonstrated marginal and inconsistent associations with these traits, Paranormal Involvement (PI) exhibited a more stable and robust relationship with Wellbeing and Hope. These distinct correlational patterns justified the use of Canonical Correlation Analysis (CCA) as a tool for further exploring the latent structures underlying variable sets.

CCA evaluated the multivariate relationship between paranormal-based variables (PI, NAP, and TPB) and indicators of positive psychological functioning (Hope Pathways and Agency, Optimism, Mental Toughness, Positive Emotion, and Eudaimonic and Hedonic Wellbeing). The full model was statistically significant, Wilks’ *Λ* = 0.85, *F*(21, 5746.34) = 15.49, *p* < 0.001. This specified that the paranormal predictors shared a sizeable portion of variance with the psychological outcomes. The overall multivariate effect size, calculated as 1 − Wilks’ Λ, was 0.15, indicating that the combined canonical model accounted for 15% of variance shared between the two variable sets. The squared canonical correlations for individual functions ranged from 0.01 to 0.11. The first and second significant functions accounted for 11 and 5% of shared variance, respectively, representing modest canonical effects.

Dimension reduction analysis confirmed that the first function (capturing roots one to three) was significant. The second function also reached statistical significance, *F* (12, 4004.01) = 8.62, *p* = 0.001. The remaining function was not significant. Significant functions accounted for 10 and 5% of the data variance, respectively. Standardized canonical structure coefficients appear in Table 2. Following established guidelines, analysis designated variables with structure coefficients exceeding 0.30 as primary contributors to their respective functions. To ensure conceptual clarity, where a variable loaded above 0.30 on both functions, it was assigned to the function on which it demonstrated the greatest loading.

**Table 2:** Structure coefficients (canonical loadings) for significant canonical functions.

Variable	Function 1	Function 2
	*r_s_*	*r_s_*
Criterion variable set		
New age philosophy	0.92	0.34
Traditional paranormal belief	0.93	0.03
Paranormal involvement	0.56	−0.69
Predictor variable set		
Optimism	0.62	−0.20
Hope pathways	0.32	−0.61
Hope agency	0.33	−0.22
Eudaimonic wellbeing	0.30	−0.53
Positive emotion	0.23	−0.25
Hedonic wellbeing	−0.03	−0.68
Mental toughness	−0.18	−0.33

Structure coefficients (r_s_) contributing most to each function and greater than 0.3 are underlined.

TPB and NAP (see Table 2) contributed most to the criterion variable for Function 1, whereas Optimism and Hope Agency primarily characterized the predictor set. Consequently, this function was defined as PB endorsement. In contrast, PI loaded strongly on the synthetic criterion variable for Function 2, with Hedonic Wellbeing, Hope Pathways, Eudaimonic Wellbeing, and Mental Toughness emerging as the primary contributors to the predictor set.

Since canonical signs are arbitrary, the alignment of variables indicated that PI, Wellbeing, Hope Pathways, and Mental Toughness clustered together and formed a psychological profile distinct from ideological PB endorsement. Accordingly, this second function was labelled Experiential Paranormal Engagement, to reflect behavioural engagement rather than belief endorsement. These results suggested functional divergence: while endorsement was only marginally linked to proactive traits, PI was associated with a more robust psychological profile characterized by higher levels of wellbeing, hope, and mental resilience.

## Discussion

Consistent with H1, the first canonical function, defined primarily by TPB and NAP, was positively associated with Optimism and Hope Agency (with weaker contributions from Eudaimonic Wellbeing and Hope Pathways). This indicated that PB aligned with motivational–attitudinal aspects of positive psychological functioning, particularly goal-directed determination and perceived agency. Although zero-order correlations showed small and occasionally negative associations with Hedonic Wellbeing and Mental Toughness, when considered as part of a broader psychological system, PB demonstrated alignment with motivational–attitudinal traits within the multivariate system.

Supporting H3, the second function characterised by PI, demonstrated stronger associations with Hedonic and Eudaimonic Wellbeing, Hope Pathways, and Mental Toughness. This configuration was consistent with a proactive and resilient psychological profile, suggesting that these beliefs and behaviours are associated with adaptive responses to life stressors (e.g., Dudley, 2000; Rogers et al., 2006). Unlike PB, PI clustered with the capacity to generate goal-directed routes (pathways thinking) and traits reflecting confidence, persistence, and adaptive challenge appraisal.

The inclusion of hedonic and eudaimonic wellbeing further signified that PI related not only to happiness, but also to meaning, growth, and effective functioning, core components of psychological wellbeing (Ryff, 1989). This multivariate configuration was consistent with the theoretical proposition that involvement reflects experiential reinforcement, potentially being associated with agency, mastery, and existential coherence. Rather than aligning with vulnerability indicators, PI clustered with planning, resilience-related traits, and indicators of flourishing (Keyes, 2002). The absence of a negative association with Mental Toughness challenges deficit-based interpretations of PI and suggested compatibility with psychological hardiness (Clough et al., 2002; Drinkwater et al., 2019).

These outcomes demonstrate that PB and PI represent functionally distinct yet complementary components of individuals’ broader orientation toward the supernatural. Considered jointly, these dimensions may be usefully conceptualised as paranormal salience (PS). This nomenclature denotes the personal significance attributed to paranormal phenomena through both credence (belief) and engagement (involvement). Importantly, within the present study, PS is proposed as a theoretical higher-order framework rather than an empirically established latent construct. Future research should examine this proposition through formal measurement modelling, including confirmatory factor analysis and structural equation modelling. In this context, PS is not proposed as an alternative to established measures of paranormal endorsement; rather, it represents a conceptual framework for integrating belief and behavioural engagement when examining psychological correlates.

Moreover, findings indicate that PB and PI align differentially with positive psychological traits and do not uniformly correspond with deficit-based interpretations. Although TPB and NAP correlated strongly at the bivariate level, when modelled simultaneously with PI, the constructs contributed to different synthetic variates. Importantly, despite strong bivariate overlap, CCA demonstrated that TPB and NAP contributed differentially to synthetic variates when modelled alongside behavioural involvement, supporting their functional distinctiveness within multivariate structure.

PI contributed to a distinct canonical function that loaded strongly on Hedonic and Eudaimonic Wellbeing, stronger Hope Pathways, and greater Mental Toughness. This divergence was theoretically important because it validated the distinction between ideological commitment to supernatural propositions (PB) and active participation in paranormal practices (PI), supporting H2. Furthermore, consistent with H4, the presence of two significant functions indicated that PB and PI do not operate as a homogeneous psychological dimension. Instead, as evidenced by differentiated patterns of covariation, they reflect discrete configurations of belief, behaviour, and adaptive traits. Collectively, findings substantiated the proposition that PB and PI represent separate psychological configurations that jointly contribute to form PS.

Theoretically, this outcome reinforces the importance of considering whether total scores alone sufficiently capture the heterogeneous and functionally divergent nature of PB. When modelled using TPB and NAP dimensions, PB reflects a worldview orientation linked to motivational expectancy (optimism) and volitional drive (agency). This interpretation is consistent with the notion that NAP is associated with subjective meaning and perceived control and suggests that even TPB may coexist with adaptive motivational schemas (Dagnall et al., 2025a). Notably, however, this function remained primarily attitudinal, showing limited integration with Mental Toughness or broader wellbeing. PB therefore operates primarily at a cognitive–ideological level rather than as an indicator of embodied resilience.

These results provide empirical support for the premise that supernatural credence is multidimensional. Particularly that PS comprises: (1) PB, ideological endorsement associated with motivational outlook; and (2) PI, experiential engagement associated with wellbeing and resilience. This functional divergence supports the value of employing the two-factor Rasch model alongside broader assessments of paranormal endorsement and underscores the utility of CCA for modelling complex interrelations among belief systems and psychological traits. While zero-order correlations appeared weak and inconsistent, the multivariate approach revealed coherent and theoretically meaningful patterns that would have remained obscured under univariate analysis.

From a broader perspective, the findings contribute to a reframing of supernatural credence within positive psychology (Dagnall et al., 2025b; Irwin and Watt, 2007; Rogers et al., 2006; Seligman, 2012). Paranormal engagement (PI), particularly when operationalised as behavioural involvement, is compatible with adaptive traits. This does not imply that all forms of belief are uniformly beneficial, nor does it negate evidence linking belief domains with distress in specific contexts. Rather, it highlights that supernatural worldviews can coexist with, and in instances align with, proactive psychological functioning.

This interpretation is consistent with the prominent levels of PB and PI reported in modern Western societies (see Dagnall et al., 2016). By demonstrating that supernatural orientation is not inherently synonymous with psychological deficit, these results suggest that PS may represent a functionally significant framework for some individuals. Rather than viewing such worldviews as inherently maladaptive (see Goulding, 2005; Schumaker, 1987), positive psychology may benefit from examining whether PS corresponds with existential coherence, goal-directed agency, and resilience-related characteristics in contemporary life.

### Limitations

Despite advancing theoretical understanding of PB and PI, this investigation was subject to methodological constraints that limit the generalisation of findings. Specifically, the use of a cross-sectional design prevented causal inferences about relationships between paranormal and wellbeing factors. Hence, it remains unclear whether PI is associated with subsequent improvements in wellbeing and resilience-related characteristics, or whether individuals with higher levels of psychological hardiness are more likely to engage with paranormal experiences. In the context of this paper, however, establishing foundational associations was a necessary prerequisite for progression to complex modelling. Accordingly, future research should adopt longitudinal or experimental designs to establish directionality and determine if supernatural engagement functions as a sustained psychological buffer over time.

Although the canonical relationships were modest in magnitude (with individual functions accounting for 5% and 11% of shared variance), effect sizes of this nature are common within individual differences and positive psychology research, where psychological outcomes are typically influenced by multiple interacting factors. Accordingly, the present findings should not be interpreted as suggesting that paranormal involvement is a dominant determinant of wellbeing. Rather, they indicate that behavioural engagement with the paranormal is compatible with positive psychological characteristics and contributes to a broader constellation of factors associated with positive functioning.

Moreover, because measures were self-reported and administered concurrently, the findings remain susceptible to common method bias (CMB) and uniform responding. Although procedural controls were employed (e.g., scale item randomisation, assuring anonymity, and emphasizing construct independence to reduce method variance; Podsakoff et al., 2003), cross-sectional self-reports may artificially inflate shared variance. The presence of distinct, bifurcated relationship patterns across canonical functions, where specific predictors showed divergent and inverse associations with outcome variables, suggests that CMB did not account for the observed multivariate structure. Nevertheless, future research should incorporate multi-wave longitudinal designs or objective behavioural markers alongside self-report measures to further evaluate method variance and determine whether observed associations generalize beyond self-reported experiences.

PI was measured using a three-item index that captured the breadth of engagement but failed to distinguish between the intensity, frequency, and nature of experiences. Thus, the current data cannot determine whether the observed psychological benefits stem from a single, transformative experience or habitual paranormal practices. Acknowledging this, future studies should incorporate these factors into the measurement of experiential engagement. This added sophistication will provide a fine-grained, nuanced understanding of how experiential differences interact with PB and influence wellbeing, clarifying whether frequent engagement reinforces resilience more effectively than sporadic belief-based interactions.

The strong positive correlation between TPB and NAP designates that the two factors share considerable variance and conceptual overlap (i.e., items from the Witchcraft and Precognition subscales). To strengthen discriminant validity and theoretical precision researchers should further examine and enhance belief measurement and classification.

Since the sample comprised a UK-based population this may limit the generalizability of the findings to other societies. Furthermore, although participants were recruited from across the UK, the sample was not intended to be nationally representative. Consequently, the findings should be interpreted as reflecting relationships within this sample rather than estimates of population prevalence. Replication using nationally representative samples would strengthen confidence in the generalisability of the observed associations.

In addition to undertaking cross cultural comparison future research should investigate functional drivers within diverse or high-stress populations. This would determine if the positive features associated with PS extend to other socio-cultural environments.

## Conclusion

Paranormal belief (PB) and involvement (PI) together reflect a multidimensional configuration of paranormal orientation/salience (PS). Commensurate with this operationalisation, this study identified two distinct multivariate functions. Function 1 (Belief Endorsement) aligned with motivational traits. Function 2 (Experiential Engagement), clustered with Hedonic and Eudaimonic Wellbeing, Hope Pathways, and Mental Toughness. These findings highlight that supernatural worldviews can coexist with positive psychological functioning and, in multivariate context, align with motivational and resilience-related traits.

## Data Availability

The raw data supporting the conclusions of this article will be made available by the authors, without undue reservation.
